# Study on Flexural Behavior of Glass Fiber Reinforced Plastic Sandwich Composites Using Liquid Thermoplastic Resin

**DOI:** 10.3390/polym14194045

**Published:** 2022-09-27

**Authors:** Hassan Alshahrani, Azzam Ahmed

**Affiliations:** 1 Department of Mechanical Engineering, College of Engineering, Najran University, Najran 11001, Saudi Arabia; 2 Department of the Textile Engineering, College of Engineering and Technology of Industries, Sudan University of Science and Technology, Khartoum P.O. Box 407, Sudan; 3 Safat College of Science and Technology, Khartoum P.O. Box 321, Sudan

**Keywords:** GFRP, liquid thermoplastic resin, vacuum infusion, sandwich structures, three-point bending, finite element analysis

## Abstract

Experimental and numerical studies of composite sandwich structures are warranted to reap the benefits of these materials when they are well designed. In the current research, new liquid thermoplastic and epoxy resins were used to fabricate four composite sandwich panels with two additional foam types and different densities in the wind turbine industry. A comprehensive comparison of three-point bending test results was made. Finite-element-based simulations using the ABAQUS program with Hashin’s damage criterion were conducted to examine the failure behavior of the GFRP sandwich composites. The flexural behavior of the glass-fiber-reinforced plastic (GFRP) sandwich panels was investigated and compared with the experiments. The results show that the GF/PVC/Elium composite panel gives the highest load absorption, flexural strength, flexural modulus, core shear ultimate strength, and facing stress due to effect of the core foam and resin types. For the PVC foam core sandwich panel, using thermoplastic resin increased the flexural strength by 18% compared to that of the epoxy resin. The simulation results show excellent agreement between the finite-element-predicted failure loads and the experimental results.

## 1. Introduction

In recent years, composite sandwich structures have been widely used in huge applications due to their lightweight structures, good specific stiffness, strength, ability to absorb energy, resistance to corrosion, and high flexural behavior. These features make them ideal for use in the wind energy field. Glass fiber composites offer flexible processing and excellent sturdiness, and they are lightweight. In the 21st century, fiberglass utilization expanded significantly, mainly due to the increased use of wind blades and nacelles. Typical sandwich structures are divided into laminates, such as skin materials used in the top face sheet and bottom face sheet, and low-density foam, such as core and adhesives. Many studies in the field of sandwich structures have been conducted to assess the performance of their mechanical properties, both with and without simulations, and to understand the complex interactions between different composite materials. Moreover, several studies have demonstrated the behavior and failure modes of composite sandwich structures under bending load by considering the following factors: reinforcement materials [1,2,3], skin stacking sequences [4], foam types [5,6,7,8,9,10,11], adhesive types, and manufacturing conditions. Regarding high-performance applications, composite sandwich structures have been used in aeronautical materials [12], sailboat hulls [2,5], wind turbine blades [11,13,14], marine applications [15], and aerospace engineering [16,17,18]. As clean and renewable energy, wind energy has been paid increasingly more attention by countries worldwide [19]. Wind turbine blades are thin-shell structures made of composite materials [20]. There have been successful experiences of using a “sandwich” structure for these thin-shell structures at home and abroad. Most use glass-fiber-reinforced epoxy resin, with BALSA wood and PVC foam sandwiched in between [21]. Innovative geometrically modified auxetic foam (GMAF) structures were developed for impact mitigation by Asad [22]. A novel cement-based auxetic foam composite was developed by Fan et al. [23]. The results showed that the compressive strength of auxetic foam increases with an increase in foam hole density. The shear modulus of open-cell polyurethane thermoformed auxetic foams has been determined in three- and four-point bending tests. Auxetic foam has a shear modulus that is 7% lower than that of conventional bulk specimens [24].

The bending behavior of an innovative fiber-reinforced polymer (FRP) sandwich panel was investigated for application in a multistory building. The results showed that the bending stiffness and ultimate bending strength could be enhanced by increasing the web thickness, web height, and face sheet thickness, as well as by reducing the web spacing [25]. Common core materials, such as foam and honeycomb, have been widely used in sandwich structures in engineering, especially in aerospace and marine engineering [26,27,28].

PVC core material is made of rigid foam with unique foaming technology; it is light and has good stiffness and long-term fatigue resistance [29]. The commonly used densities in wind turbine blades are 60 kg/m^3^ and 80 kg/m^3^. Besides the flexural strength of the sandwich composites used for wind turbines, several studies have been conducted to assess the interface toughness (face/core debonding) using mode I or II debonding tests on a sandwich plate [30,31]. A comprehensive review on sandwich composites and their applications was carried out by Ramnath et al. [32]. The aim of the review was to identify a new method and materials for sandwich core materials for all suitable applications of composite structures with cost effectiveness suitable for the next advancement in the field. Selecting H-60 PVC foam, four-axis E-glass non-woven fabric, and vinyl resin, a type of innovative reinforced sandwich composite similar to a grooved perforation sandwich (GPS) was fabricated by VIMP. In flexural testing, the face yield and the core shear failure of the sandwich beams could be observed [33]. An evaluation of the flexural characteristics of CFRP sandwich structures with reinforcement webs was carried out by Bao et al. [34]. The results confirmed that CFRP sandwich structures with reinforcement webs have improved flexural strength. 

In a simulation analysis, composite sandwich structures made of different skin materials (Kevlar, glass, and carbon fibers) and foams were experimentally and numerically evaluated using various simulation software programs [3]. The quasi-static three-point bending behavior of different stacking sequences of the skin materials of sandwich structures was evaluated in a simulation. The results of the load–displacement curves demonstrated that the experimental and numerical models were in good agreement [4]. The numerical model investigated the quasi-static three-point bending of CFRP sandwich beams with square honeycomb cores. Furthermore, the finite element model (FEM) predicted that each composite specimen had a measurable load–displacement response and failure progress [6]. Sun et al. conducted a numerical study to design a method for evaluating the flexural strength and continuum damage of composites with different graded corrugated truss cores using ABAQUS software. According to the simulation results, the considered structure with the graded core width designed in a lower convex function arrangement will achieve a higher buckling load under a three-point bending condition [7]. The results of three-point bending tests applied to four different types of composite sandwich structures were compared to those of an FEM. The experimental and FEM results showed that the sandwich behavior is affected by skin and foam thicknesses [13]. Experimental, theoretical, and numerical investigations were conducted on a composite sandwich structure’s bending load and failure mode. Sandwich structures were fabricated using a glass-fiber-reinforced polymer (GFRP) as the face sheet, while polyvinylchloride (PVC) foam was used as the core material. The results showed that core shear failure associated with skin–core debonding occurred close to the loading points [15]. Experimental and numerical studies on the flexural behavior of composite sandwich structures manufactured using the VARTM process were conducted. The produced sandwich structures were subjected to three- and four-point bending loads. The results showed that the experiments and simulation were in excellent agreement [35]. Moreover, an experimental investigation and a numerical study were carried out on the flexural behavior of sandwich panels made of aluminum skins and a Nomex™ honeycomb core. Comparisons between the experiments and numerical models were made using load–displacement curves and were further illustrated using photographic images. The FEM numerical results gave a clear indication of the damage to the structure [36]. The dynamic flexural behavior of sandwich beams with composite face sheets and a foam core was analyzed by developing a 3D FEM. Comparisons between the experimental and numerical models showed that the results were acceptable in terms of contact force histories, peak force values, absorbed energy, and the maximum displacement of both face sheets [37]. Static behavior and finite element modeling analyses were conducted to investigate face sheet thickness and core thickness effects using ANSYS Workbench software. The results concluded that core and skin thicknesses are related to equivalent maximum stress, shear stress, and deformation [38]. A finite element analysis (FEA) explains crack initiation and propagation under flexural load, and a progressive damage method can be used to simulate the failure of a foam core sandwich structure [39]. Furthermore, an analysis on a composite sandwich structure was conducted using the strain-energy-based homogenization method. The bend test results for both analyses were compared with experimental data and were in good agreement [40]. 

The main benefits in the wind turbine industry were acquired by studying and understanding the behavior of new sandwich composite structures under flexural load and by using different resin materials, such as liquid thermoplastic resin rather than thermoset resin. Therefore, this study investigates the effects of various resins (Epoxy 2040 and Elium 188) and foams (PVC and PET) on the flexural strength properties of the glass-fiber-reinforced plastic sandwich composites used for wind turbine blades. Moreover, the current study determines the influence of process parameters, namely, different resin materials, core types, and densities, on the sandwich composite flexural behavior. An FEM of the flexural behavior of the sandwich composite structures is implemented using ABAQUS software. 

## 2. Materials and Testing

### 2.1. Skin Materials

A glass fiber plain weave was used as the face sheet/skin with high specific strength and stiffness. The fabric weight was 227 g/m^2^. The upper and lower skins were two-ply, each with the same stacking sequence. To determine the required mechanical properties (which are shown in Table 1) of the laminates used in the facings of the sandwich material, the rule of mixture equations and tensile tests were performed according to the ASTM-D3039 for GF/Epoxy and GF/Elium sandwich composites. The mechanical properties presented in Table 1 are important data for performing the finite element modeling of flexural tests with ABAQUS software.

### 2.2. Core Materials

In this study, two types of foam were used: First, Gurit^®^ PVC is a closed-cell, cross-linked PVC foam. For other high-performance composite applications, it has an excellent strength-to-weight ratio, excellent thermal insulation capabilities, negligible water absorption, and outstanding chemical resistance. Second, Gurit^®^ G-PET™ is a highly versatile, recyclable, thermoplastic core material with a good balance between mechanical properties, temperature resistance, density, and cost for applications and processes. It can be used with numerous resin systems, including epoxy, vinyl ester, unsaturated polyester, and phenolic resins, according to the ASTM D1621-10 standard [41]. The mechanical properties of the foams are presented in Table 2.

### 2.3. Liquid Thermoplastic Resin

Elium^®^ 188 is a low-viscous liquid thermoplastic resin suitable for vacuum infusion. This new resin can be used to manufacture glass fiber, carbon fiber, and natural fiber-reinforced thermoplastic composites using low-pressure processing technology and molds that are widely used today for unsaturated polyesters and epoxy resins. Molded thermoplastic composite parts possess mechanical properties comparable to those of epoxy composites. Their main advantages are their thermoplastic shape and recyclability and that they provide an assembly bond between composites and composites or between composites and metals. The manufacturing properties of Elium^®^ 188 resin compared with those of Epoxy 2040 resin are presented in Table 3. 

### 2.4. Fabrication Process

In this study, the core material chosen for the sandwich composite structures is closed-cell PVC and PET foams with densities of 60 and 75 kg/m^3^, respectively. In the current study, the thickness of the foam core is 20 mm. The foam is located between two layers of GFRPs (upper face sheet, foam, and lower face sheet), each composed of two layers (±45)_2_ of plain weave E-glass. Two types of resin are used in a 5:1 resin-to-hardener ratio for the resin infusion. The sandwich panels are manufactured using the vacuum infusion technique. The panels are cured at room temperature (20 °C and 25 °C) for six hours with humidity between 50 and 70% and consolidated under uniform atmospheric pressure.

### 2.5. Flexural Test

**Figure 1:**
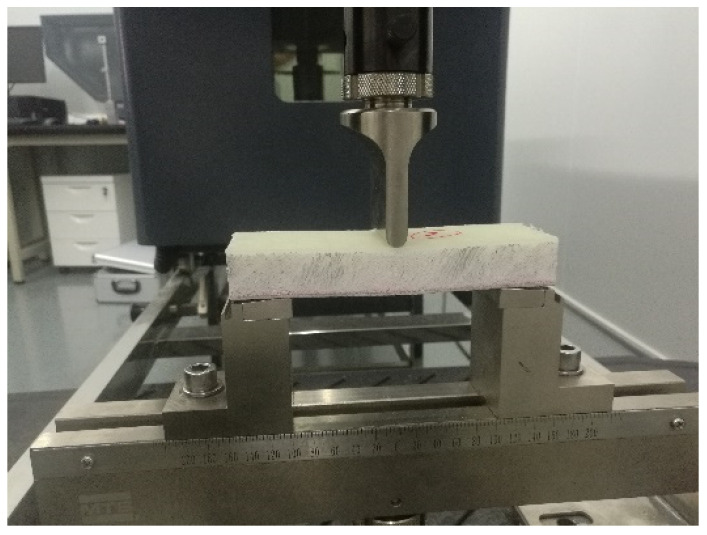
Three-point bending test with sample.

Flexural tests were carried out using a three-point bending test according to ASTM C393. Figure 1 illustrates the three-point bending device (a Universal Testing Machine MTS^®^ equipped with a 100 kN load cell). The radius of the semi-spherical indenter was 10 mm. At least three specimens for each sandwich composite structure were measured. The applied velocity was 5 mm/min. Water jet machining was used to cut the bending test specimens. The length, width, thickness, and span length measurements were 150, 50, 24, and 100 mm, respectively. The samples were tested until failure occurred, and then the flexural strength of the sandwich composites was calculated according to the formulations of ASTM D790-90 for various types of sandwich structures. The core shear strength and the facing stress of the sandwich composites were calculated according to ASTM C393.

## 3. Finite Element Modeling

### 3.1. Modeling of the Foam

**Figure 2:**
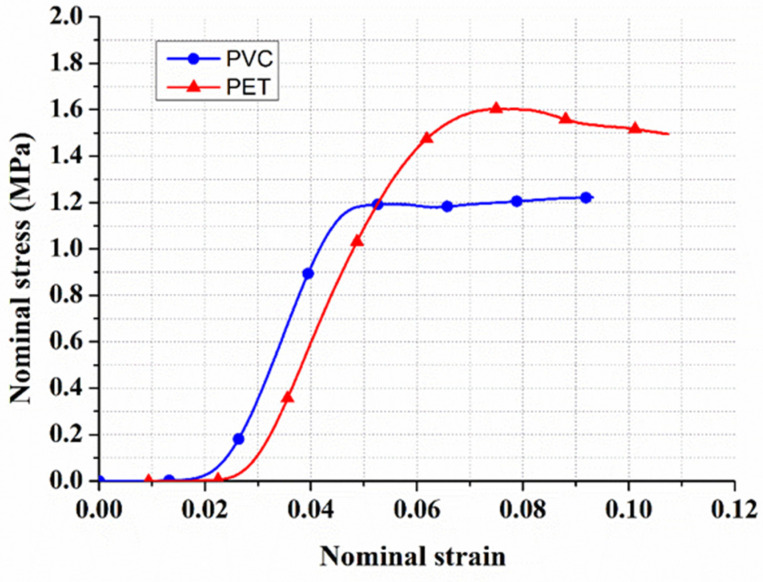
Nominal stress–strain curve for a compression test of PVC and PET foam.

As a crushable foam of an elastic–plastic material, the core was modeled using hardening curves obtained from conducting uniaxial compression tests on square blocks. The elastic part of the foam was defined as an isotropic elastic option with two parameters: Young’s modulus and Poisson’s ratio. As shown in Figure 2, compression test curves were obtained to describe the crushable foam plasticity behavior [42]. The foam part was described as a solid shape in ABAQUS/Explicit. The element type of the foam was C3D8R, and 18,750 was the number of elements.

### 3.2. Modeling of the Upper and Lower Skins

The upper and lower skins were modeled as an elastic material, with Glass/Epoxy and Glass/Elium, as presented in Table 1. The continuum shell section was used to define the structure of the upper and lower skins, the material type and thickness, and the orientation of each layer. The upper and lower face number of elements was 15,000. The “Tie” element was used for the upper and lower skins attached to the foam, and tangential and normal behavior was the interaction property between surfaces. Hashin’s failure criterion was used to predict the damage initiation in the upper skin, including four damage initiation mechanisms to detect the matrix and fiber under tension and compression failures [43,44,45]. The effect of Hashin’s damage can be seen when one of these failure modes occurs. The failure modes included in Hashin’s criterion are as follows:

1. Tensile fiber failure for *σ*_11_ ≥ 0:

$${\left(\frac{\sigma_{11}}{X_{T}}\right)}^{2}+\frac{\sigma_{12}^{2}+\sigma_{13}^{2}}{S_{12}^{2}}=\left\{\begin{array}{c} \geq 1\;\mathrm{failure}\\ <1\;\mathrm{no}\;\mathrm{failure} \end{array}\right.$$

2. Compressive fiber failure for *σ*_11_ < 0:

$${\left(\frac{\sigma_{11}}{X_{C}}\right)}^{2}=\left\{\begin{array}{c} \geq 1\;\mathrm{failure}\\ <1\;\mathrm{no}\;\mathrm{failure} \end{array}\right.$$

3. Tensile matrix failure for *σ*_22_ + *σ*_33_ > 0:

$$\frac{{\left(\sigma_{22}+\sigma_{33}\right)}^{2}}{Y_{T}^{2}}+\frac{\sigma_{23}^{2}-\sigma_{22}\sigma_{33}}{S_{23}^{2}}+\frac{\sigma_{12}^{2}+\sigma_{{}_{13}}^{2}}{S_{12}^{2}}=\left\{\begin{array}{c} \geq 1\;\mathrm{failure}\\ <1\;\mathrm{no}\;\mathrm{failure} \end{array}\right.$$

4. Compressive matrix failure for *σ*_22_ + *σ*_33_ < 0:

$$\left[{\left(\frac{Y_{C}}{2S_{23}}\right)}^{2}-1\right]\left(\frac{\sigma_{22}+\sigma_{33}}{Y_{C}}\right)+\frac{{\left(\sigma_{22}+\sigma_{33}\right)}^{2}}{4S_{23}^{2}}+\frac{\sigma_{23}^{2}-\sigma_{22}\sigma_{33}}{S_{23}^{2}}+\frac{\sigma_{12}^{2}+\sigma_{{}_{13}}^{2}}{S_{12}^{2}}=\left\{\begin{array}{c} \geq 1\;\mathrm{failure}\\ <1\;\mathrm{no}\;\mathrm{failure} \end{array}\right.$$

5. Interlaminar tensile failure for *σ*_33_ > 0:

$${\left(\frac{\sigma_{33}}{Z_{T}}\right)}^{2}=\left\{\begin{array}{c} \geq 1\;\mathrm{failure}\\ <1\;\mathrm{no}\;\mathrm{failure} \end{array}\right.$$

6. Interlaminar compression failure for *σ*_33_ < 0:
  $${\left(\frac{\sigma_{33}}{Z_{C}}\right)}^{2}=\left\{\begin{array}{c} \geq 1\;\mathrm{failure}\\ <1\;\mathrm{no}\;\mathrm{failure} \end{array}\right.$$
  where *σ_ij_* denotes the stress components, and the tensile and compressive allowable strengths for the lamina are denoted by subscripts *T* and *C*, respectively. *X_T_*, *Y_T_*, and *Z_T_* denote the allowable tensile strengths in the three respective material directions. Similarly, *X_C_*, *Y_C_*, and *Z_C_* denote the allowable tensile strengths in the three respective material directions. Furthermore, *S*_12_, *S*_13_, and *S*_23_ denote the allowable shear strengths in the respective principal material directions.

### 3.3. Flexural Test Model

**Figure 3:**
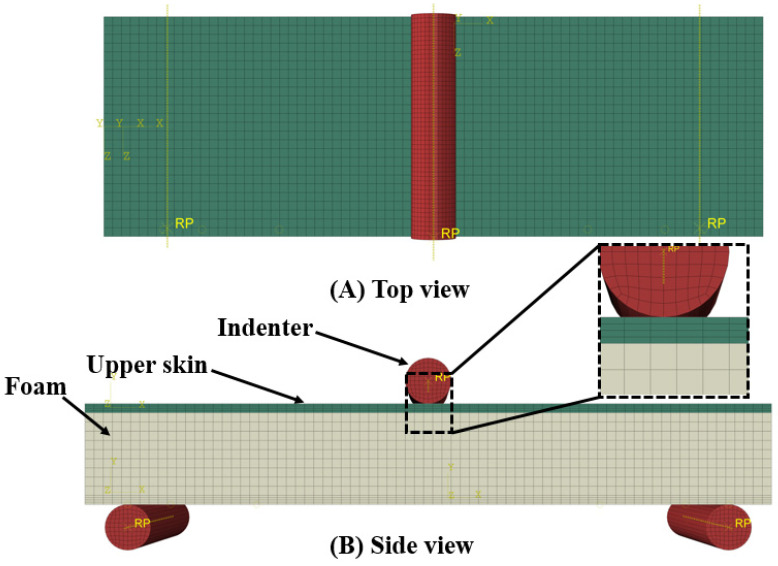
Three-point bending test system.

According to the ASTM C393 testing conditions, a three-point bending testing geometrical model was created, as shown in Figure 3. A graded mesh was created, and finely meshed was made in the region of the impact surface of the upper skin. An SC8R continuum shell was used to model the upper and lower skins. As a rigid body, the indenter was defined relative to the sandwich panel with a surface-to-surface contact interaction with normal and tangential behavior. The number of elements of the indenter was 2792. General static steps were defined with specific dissipated energy fractions.

## 4. Results and Discussion

### 4.1. Experimental Results 

**Figure 4:**
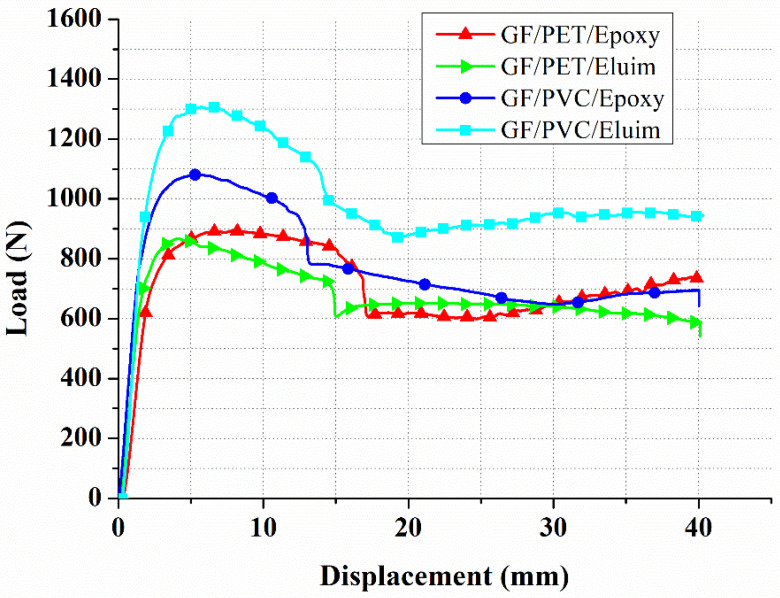
Load vs. displacement of GFRP sandwich composites.

Figure 4 and Table 4 show a load versus displacement graph, and the flexural strength and modulus of the various prepared GFRP composites. It was noted that the GF/PVC/Elium GFRP composite panel gives the highest load absorption of 1308 N at the significant displacement of 5.3 mm, which is equal to a flexural strength and modulus of 243.75 MPa and 0.26 GPa, respectively. However, the other combinations also offer an effective load absorption, flexural strength, and flexural modulus. The observed loads of 1156 N, 868 N, and 845 N, which are equal to flexural strengths of 206.25, 165, and 159.375 MPa and flexural moduli of 0.215, 0.18, and 0.16 GPa were noted for GF/PVC/Epoxy, GF/PET/Elium, and GF/PET/Epoxy, respectively. This higher load-bearing effect is the reason for the higher strengthening effect of the PVC core structure. The load applied during the bending process is absorbed by the core unit, which contains the novel structures of PVC and PET. It was noted that the PVC core provided a higher reinforcement than the PET among all the composites. This is because of the incredibly soft nature of the PVC molecular structure than the PET. Since the PET is semi-crystalline or amorphous based on the curing condition, the molecules are rigid, with no free volume to stretch. Thus, the PET core sandwich panels produced lower load absorption, whereas, in the PVC, the load could transfer uniformly without crack propagation and cleavage formation. However, the PVC core unit contains composites that offer a higher flexural strength. PVC is robust and linear. The monomers are primarily head-to-tail, resulting in chlorides on alternate carbon centers. PVC has a mostly atactic stereochemistry, which means that the chloride centers’ relative stereochemistry is unpredictable. A certain degree of a syndiotactic configuration in the chain results in a high degree of crystallinity, affecting higher stretching and flexibility [46]. Thus, the PVC foam structure in the core part restricts the degree of crack propagation and prevents it from growing further. Hence, the entire bending load in the three-point bending could be absorbed by the form core unit, and, therefore, the matrix was free from the stress intensity factor. The composites could withstand the higher load since the stress intensity factor was less [15]. As the same study was carried out by Herranen et al. [47], the flexural strength and stiffness of the three sandwich panels, which had similar GFRP facings and different core materials (PET, HDPE, and PMI), were determined. Four-point bending tests showed that the sandwich panel, which had a PMI foam core, achieved the best results with regard to stiffness. As compared to the highest peak load, the flexural strength and deflection values of the PET core materials were 2.1 KN, 17 MPa, and 48.2 mm. In the current study, these values for the PET core materials, such as GF/PET/Epoxy, were 0.85 KN, 159.4 MPa, and 40 mm, respectively. The effects of the elevated in-service temperature on the flexural behavior of the sandwich composites consisting of glass-fiber-reinforced polymer (GFRP) skins and a phenolic core were investigated by Manalo et al. [48]; the flexural behavior of the sandwich beams was governed by the GFRP skins up to 80 °C. The results showed that the phenolic core played a major role in the overall behavior beyond this temperature. As the behaviors of the core materials (PVC and PET) used in this study were observed to be the same, both of the materials have a significant effect on the flexural tensile properties and damage modes. The flexural properties of a novel foam core GF/PVC/Vinyl sandwich structure reinforced by stiffeners were investigated by Liu et al. [49]; the results showed that, after inserting stiffeners, the failure mode changed from indentation failure to core shear, and the ultimate load increased from 2.11 kN to 2.74 kN. In the current study, without stiffeners, the highest load was 1.3 Kn in GF/PVC/Elium. In a previous study, aluminum hybrid composite foams (AHCFs) with densities varying between 0.88 and 1.14 g/cc were fabricated, and the study revealed that the flexural strength of the AHCFs increased with their density. In the current study, various densities of the same foam were not considered, and only two types of foam with different densities were used. Some studies used different metal foam thicknesses and found that, with an increase in the foam core thickness, the flexural load carrying capacity, bending stiffness, and energy absorption capacity of the sandwich structure significantly increased [50]. Moreover, some studies considered various metal foam pore sizes and found that the stiffnesses of the composites increase with a decrease in the pore size [51]. 

It is also noted that the amount of displacement in the composites was restricted to less than 7 mm. Beyond this level, the composites start repelling the amount of load applied. This is because of reaching the elastic limit, and beyond this, fracturing begins to occur. When an oversized load is applied to the composite, the core part absorbs and slightly elongates it. Once the load reaches the tear limit of the foam, the composite cannot withstand the applied load, leading to fracture [52]. It is noted that the GFRP composite panel GF/PET/Epoxy sandwich structure gives a significantly lower flexural strength than the peer group. This is because of the highly rigid molecular structure of PET since PET comprises polymerized units of the monomer of ethylene terephthalate with repeating (C_10_H_8_O_4_) units. Based on the processing and heating history, polyethylene terephthalate may be amorphous (transparent) and semi-crystalline. Thus, the fracture occurred early in the composites and offered lower flexural strength. The retained brittleness in the PET is the cause of cleavage fracture during the bending process [53,54]. The mechanism of action of the sandwich material is to transfer shear forces from the skin layer to the inner layer so that both skin layers remain stable under static and dynamic loads and absorb shock energy in order to provide resistance to damage. The core shear properties of the sandwich composites are presented in Table 5. As can be seen in Table 5, the GF/PVC/Elium and GF/PET/Epoxy sandwich composites have the highest and lowest core shear ultimate strength, respectively, due to the strength of the core itself. Resin type has a significant effect on the core strength, which was revealed between GF/PVC/Elium and GF/PVC/Epoxy and between GF/PET/Elium and GF/PET/Epoxy when the core type was constant. Elium resin has been shown to have excellent bonding to reinforcement and foam cores. Different foam cores and the same resin (GF/PVC/Elium and GF/PET/Elium, and (GF/PVC/Epoxy and GF/PET/Epoxy) were used, and it was found that the foam core also has a significant effect on ultimate strength. The GF/PVC/Elium and GF/PET/Epoxy sandwich composites were shown to have the highest and lowest facing stress, respectively, due to the strong effect of their core foam and resin types. The polyvinyl chloride (PVC) foam with the best shear performance was used as the core material, and the bending performance of the sample was the best.

### 4.2. Validity of FEM Results

**Figure 5:**
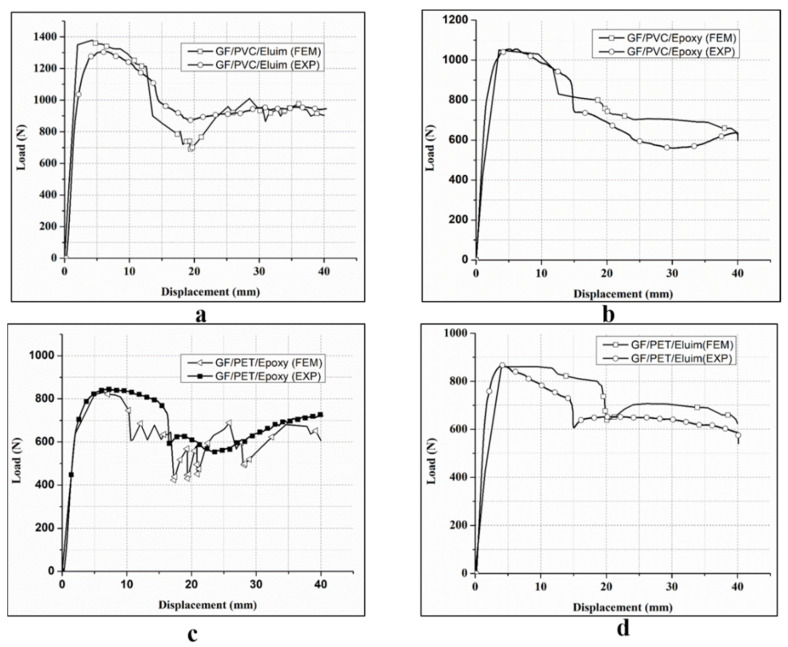
Load–displacement curves for flexural tests of GF/PVC/Elium (**a**), GF/PVC/Epoxy (**b**), GF/PET/Epoxy (**c**), and GF/PET/Elium (**d**) sandwich composites.

**Figure 6:**
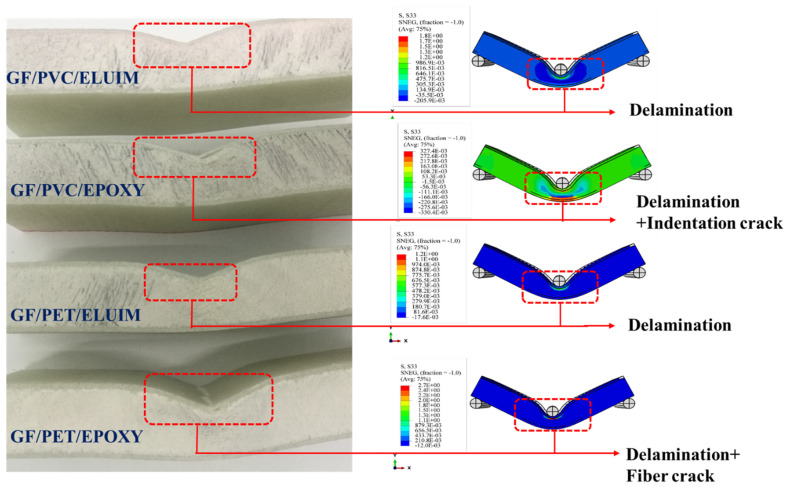
Failure behavior of the sandwich structures with different foams and resins.

A comparison of the test and the modeling of the load–displacement curves for the different sandwich structures was made to approve the FEM of the sandwich, as displayed in Figure 5. The correlations show an excellent arrangement between the test and simulation outcomes, as well as a general pattern. Each curve is comparable to the four sandwich composite design types, namely, GF/PVC/Elium, GF/PVC/Epoxy, GF/PET/Elium, and GF/PET/Epoxy, as displayed in Figure 6. The curves can be separated into three areas. The main area, linear in appearance, can clarify the variable deformation of the structure. Around here, a linear increase occurred without deformation. The deformation determines when the removal of the indenter impacted the upper skin. The second area, after the load, arrived at a peak value, and a massive drop of around 40–50% of the pinnacle load in the load–displacement was noticed for all sandwich structures; this abrupt drop is because of the foam cracking and upper skin–core interface damage. After the crack starts on the tensile side, it can spread to the compressive side of the sandwich before the final failure occurs. After the load drop, the sample supported the load to reach a critical load. The third area, with the upper skins conveying the load and the plateau region, was observed until reaching the final failure. The length of the plateau area depends on the ductility property of the foam being used.

A sudden drop in the load–displacement curve occurred due to the critical point of the structure carrying a load. In the case of the path of the curve, a good agreement between the experimental and numerical results was observed. The experimental and numerical peak loads obtained from the load–displacement curve under the different foam types were compared. An agreement was found between the experimental and finite element analysis results. The constant engineering calculations (stiffness (*E*_11_, *E*_22_, and *E*_33_), Poisson’s ratio, and the shear modulus) became an input for the simulation results. Comparing the peak loads of the foam sandwich structures in a three-point bending load test, it is evident that GV/PVC/Elium has a higher peak load than the other sandwich structures.

### 4.3. Failure Behaviors

**Figure 7:**
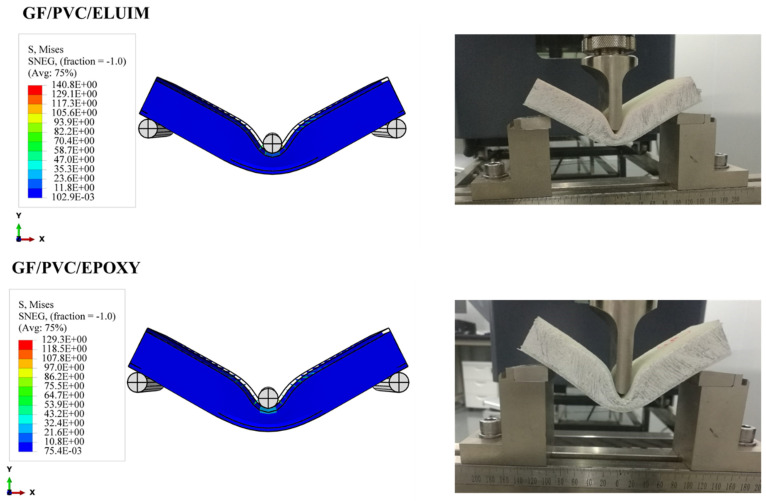
Experimental and numerical damage mechanisms under the flexural load of GF/PVC/Elium and GF/PVC/Epoxy sandwich composites.

The damage modes of the structures (GF/PVC/Elium, GF/PVC/Epoxy, GF/PET/Epoxy, and GF/PET/Elium) tested in the flexural test can be summarized as tensile failure and local indentation, followed by upper skin debonding at the impact point of the indenter. Specimen failure during the flexural test can occur due to the load transferred to the core being more than its strength at the moment of skin failure. Figure 6 shows an experimental and numerical simulation of the failure modes of the sandwich composite structures under flexural bending. The results of the FEM analysis show a satisfactory agreement with the experimental results. Comparing the failure modes of GF/PVC/Elium, GF/PVC/Epoxy, GF/PET/Elium, and GF/PET/Epoxy, all the sandwich structures have similar failure behavior, except for GF/PVC/Elium, which only shows local indentation without face sheet debonding at the impact point of the indenter. This is due to the bonding of the Elium resin with the PVC foam being stronger than that with the epoxy resin. Figure 7 shows the failure morphology (including the local indentation and face sheet damage on the tensile side and the face–core interface damage) of the foam sandwich composite under flexural loading.

#### 4.3.1. Foam Damage

**Figure 8:**
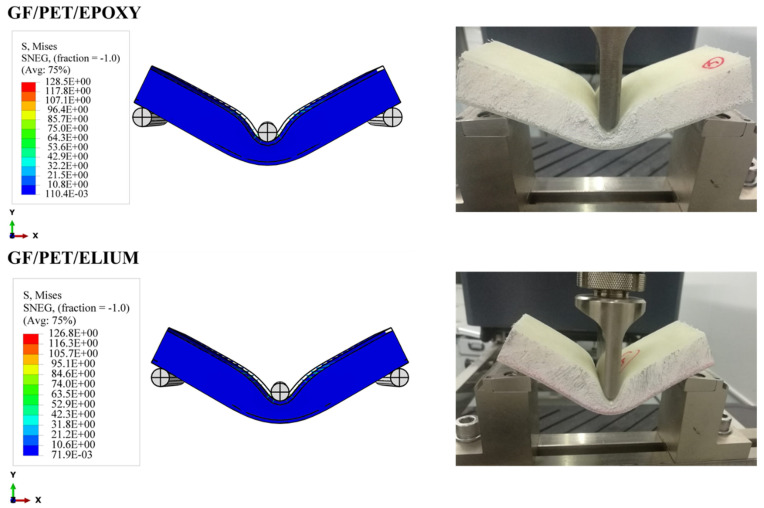
Experimental and numerical damage mechanisms under the flexural load of GF/PET/Epoxy and GF/PET/Elium Sandwich composites.

With the deepening of the application of the sandwich structures, in many cases, the sandwich structure will bear alternating loads, so the damage problem of the sandwich structure becomes greater; it becomes increasingly more prominent. The damage form can be attributed to the foam’s properties and the core material’s shear. The damage problem of the sandwich structure is more important than the other materials and structural forms used. Figure 7 and Figure 8 show comparisons between the experimental and numerical results of the damage and failure mechanisms of the GFRP sandwich composites.

As shown in several studies, the failure of a sandwich structure can occur through several damage mechanisms, such as delamination, skin–core debonding, core shear failure, skin compressive/tensile failure, core indentation failure, and core shear failure [55,56,57,58].

The predominant mechanisms in the numerical results illustrated below are local indentation, global bending, and debonding between the upper skin and the core, which are evident in the tested sandwich panel specimens. The numerical and experimental results of the sandwich panel structures are compared and analyzed for a better understanding. The difference between the experimental and numerical results in the peak load calculations for the GF/PVC/Elium specimen is 5.4%, according to the obtained data in Table 6. The most significant reason for the difference between the results is likely the neglect to the damage mechanisms across the sample thickness, including the delamination.

When the sandwich structure is bent, the sandwich structure mainly depends on the shear of the core material to transmit the force directly applied to the upper skin. In a composite sandwich structure, the modulus and strength of the upper skin are very high; only core materials with shear strength and a high shear elongation at break are suitable, such as the use of two types of foam core materials, PVC and PET. According to the possible stress conditions of the sandwich structure, the appropriate type and density of the core materials should be selected, the surface layer and core material’s thickness should be rationally designed, and the stress calculation method or the relevant finite element analysis software should be used.

#### 4.3.2. Upper and Lower Skin Damage

**Figure 9:**
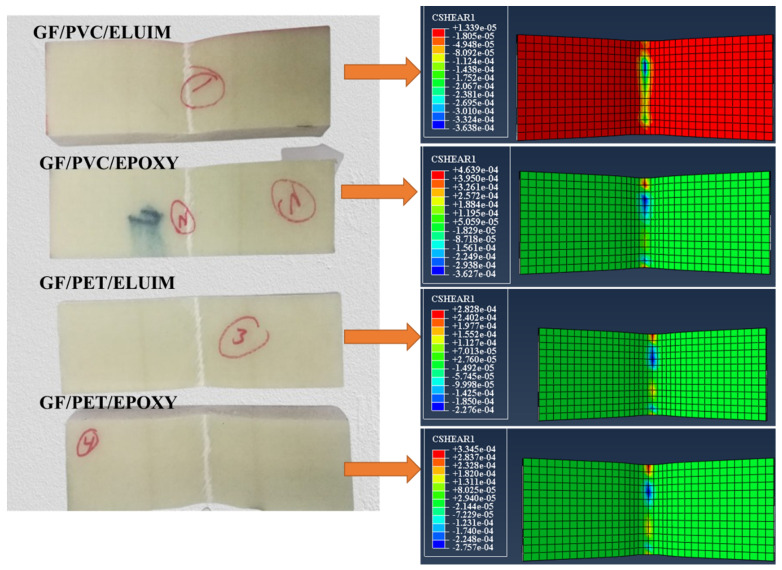
Experimental and simulation damage of the GFRP sandwich composites’ upper face sheets.

As shown in Figure 9, the shape and size of the damage that could occur to the upper skin on the tensile side depend on the stiffness of the composite sandwich structure; all types of sandwich composite structures have an almost identical failure mode: fiber breakage in the upper skins. It can be seen that the upper skin bears most of the tension and pressure, and the core material bears most of the shear force. The strength and rigidity of the upper skin are much greater than those of the sandwich material. Under bending loads, the upper and lower skins bear the principal tensile and compressive stresses, and the core material mainly delivers the shear stress. The mechanism of the core material is to form a connection with the surface layer in order to make it an integral component. A thin and robust surface layer bears a higher tensile and compressive stress without buckling, and the shear force is transmitted from the surface to the inner layer. The experimental tests show that the sandwich structures were harmed under the impact of the indenter, as shown also in the simulation photos. According to Hashin’s failure criterion, the damage criterion is used to understand the damage initiation of the specimens in the simulation case. While Hashin’s failure model has been broadly utilized in various works, it is not fit to anticipate the initiation of the matrix compressive failure mode definitively.

**Figure 10:**
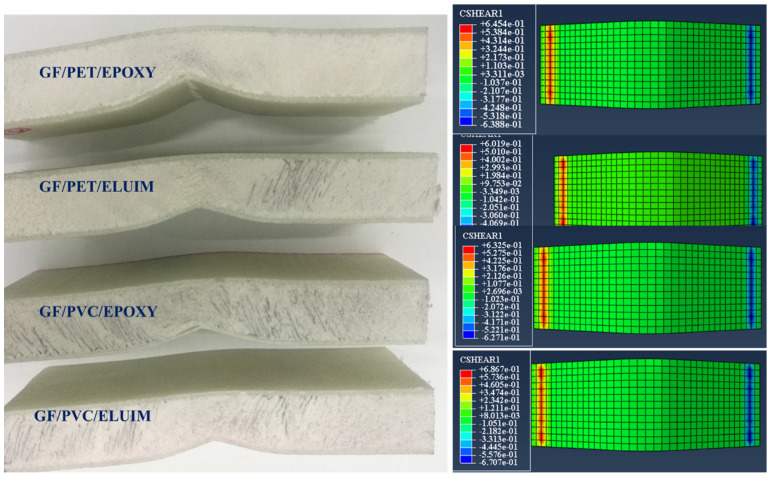
Experimental and simulation damage of the GFRP sandwich composites’ lower face sheets.

In Figure 10, lower face sheet shape damage could not be obviously observed in both the experimental and simulation results due to the high flexibility of the foam. Thus, when the core materials were subjected to the bending test, the foam returned to its original point without any damage occurring in the lower face sheet of the sandwich composites. All the failure modes and damage shapes only occurred at the impact points (in the upper face sheet, as shown in Figure 9) and through the foam thickness.

**Figure 11:**
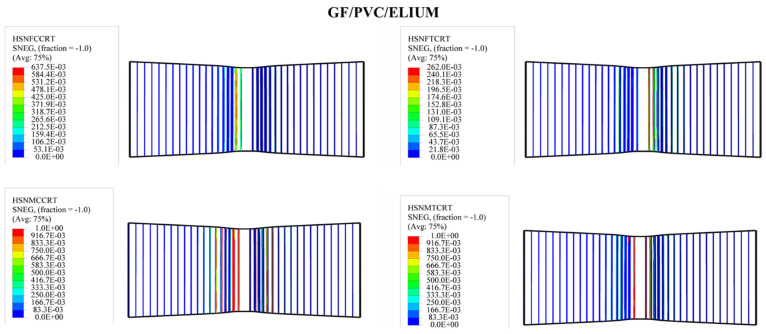
Numerical Hashin’s damage initiation of the upper skin of GF/PVC/Elium sandwich composites.

**Figure 12:**
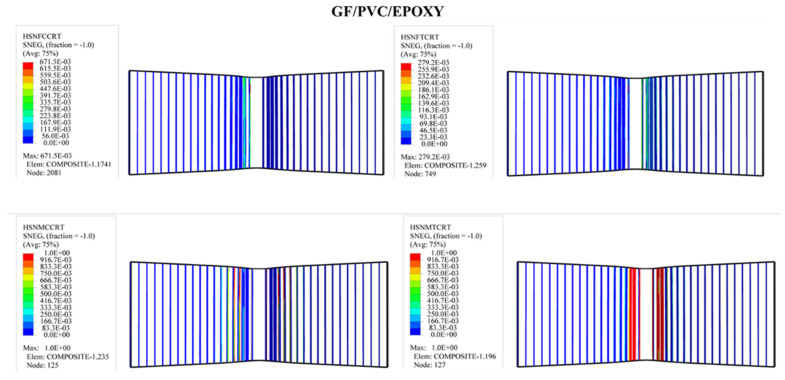
Numerical Hashin’s damage initiation of the upper skin of GF/PVC/Epoxy sandwich composites.

**Figure 13:**
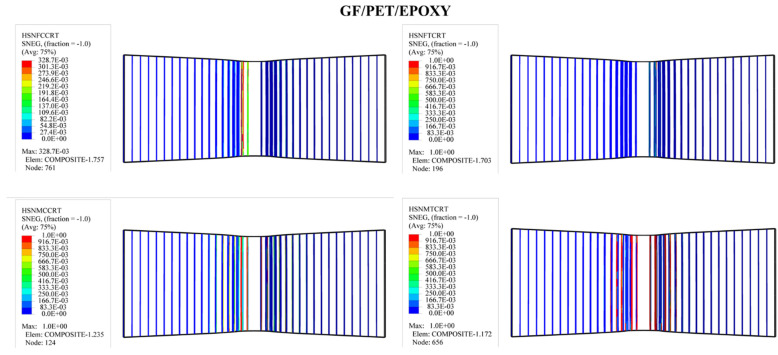
Numerical Hashin’s damage initiation of the upper skin of GF/PET/Epoxy sandwich composites.

**Figure 14:**
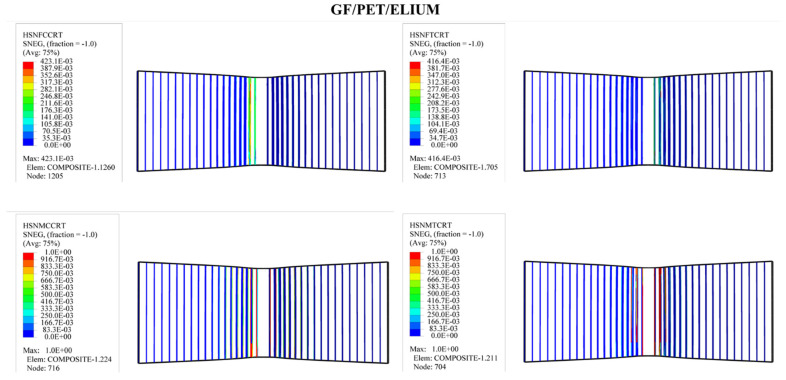
Numerical Hashin’s damage initiation of the upper skin of GF/PET/Elium sandwich composites.

In order to predict the damage caused to fiber-reinforced composites, Hashin’s damage was used for GFRP sandwich composites, as shown in Figure 11, Figure 12, Figure 13 and Figure 14. Hashin’s criterion consists of four parameters: Hashin’s fiber compressive damage initiation (HSNFCCRT), Hashin’s fiber tensile damage initiation (HSNFTCRT), Hashin’s matrix compressive damage initiation (HSNMCCRT), and Hashin’s matrix tensile damage initiation (HSNMTCRT). From Figure 11, Figure 12, Figure 13 and Figure 14, it was found that the model could predict the initiation damage due to all the values of the predicted property being less than or equal to 1. Moreover, damage was only shown to occur on the impacted side of the indenter in the middle of the specimen with different colors. 

## 5. Conclusions

The current study evaluated the flexural strength behavior of glass fiber sandwich composites with two foams (PVC and PET) and resins (liquid thermoset and thermoplastic). A finite element analysis was also conducted and validated with experimental results. Load versus displacement and flexural strength graphs of various GFRP composites were obtained. The results show that the GF/PVC/Elium composite panel gives the highest load absorption, flexural strength, flexural modulus, core shear ultimate strength, and facing stress due to effect of its core foam and resin types. The GF/PET/Epoxy sandwich structure gave a lower bearing load and flexural strength than the other structures. This is because of the highly rigid molecular structure of PET foam. The PVC foam cores achieved a better compressive strength than their PET counterparts (stiffer and more brittle). The shear performance of the GF/PVC/Elium sandwich structure was higher than that of the traditional sandwich structure. A comparison between the experimental and simulation of the load–displacement curves for the different sandwich structures was utilized to approve the FEM of the sandwich. The correlations show an excellent arrangement between the test and the simulation outcomes, as well as a general pattern of the curves’ behaviors. The failure modes in the current study can be summarized as tensile failure, local indentation, upper skin debonding at the impact point of the indenter, and delamination through the thicknesses of the sandwich composites. There was good agreement between the experimental and simulation results in terms of failure modes. When subjected to flexural loads, the type and size of the damage caused to the sandwich composites depended on the reinforcement, foam, resin properties, and test conditions. Hashin’s criterion was used in this study to predict the failure modes of the sandwich structures. After damage is initiated, the material’s stiffness gradually degenerates. At this time, it begins to enter the stage of damage evolution. Elium resin opens an interesting new field of high-performance applications for green composites and further challenges requiring more studies to be conducted on this type of liquid thermoplastic resin. It could soon be used in aerospace and renewable energy. Regarding the future considerations and recommendations for studies on renewable energy, such as those on wind turbines, the essential aspect of the research is that establishing full-scale products is the best means of producing the optimum design of thermoplastic sandwich structures using Elium resin, as this approach can solve the challenges of recyclable composites and end-of-life waste, thereby achieving huge damage and cost reductions.

## Figures and Tables

**Table 1:** The properties of skin material being used.

Property	GF/Epoxy	GF/Elium	Property	GF/Epoxy	GF/Elium
Longitudinal Young’s moduli, E_11_ (GPa)	15.1	15.6	Longitudinal tensile strength, X_t_ (MPa)	280	292
Transverse Young’s moduli, E_22_ (GPa)	15.1	15.6	Longitudinal compressive strength, X_c_ (MPa)	144.5	176
Out-of-plane Young’s moduli, E_33_ (GPa)	3.7	3.9	Transverse tensile strength, Y_t_ (MPa)	280	292
Poisson’s ratio, ν_11_	0.21	0.21	Transverse compressive strength, Y_c_ (MPa)	239	176
Poisson’s ratio, ν_13_	0.1	0.1	Longitudinal shear strength, S_12_ (MPa)	48	62
Poisson’s ratio, ν_23_	0.1	0.1	Transverse shear strength, S_23_ (MPa)	48	62
Shear modulus, G_12_ (GPa)	2.3	2.6	Density, ρ (kg/m^3^)	1624	1550
Shear modulus, G_13_ (GPa)	2.3	2.6	Thickness, h (mm)	2	2
Shear modulus, G_23_ (GPa)	1.96	2.53	Fiber volume fraction (%)	69	68

**Table 2:** Description of the properties of foam material being used.

Property	PVC	PET
Density (kg/m^3^)	60	75
Elastic modulus (MPa)	100	89
Shear modulus (MPa)	21	13
Tensile strength (MPa)	1.82	1.49
Compressive strength (MPa)	0.98	0.96
Shear strength (MPa)	0.79	0.53
Elongation at break (%)	18	44

**Table 3:** The mechanical properties of resins being used.

Property	Epoxy 2040	Elium^®^ 188
Density (kg/m^3^)	1.16	1.18
Tensile strength (MPa)	45–85	55–76
Tensile modulus (MPa)	2800–3400	3100–3300
Flexural modulus (MPa)	2600–3600	3250
Flexural strength (MPa)	100–130	130
Elongation at break (%)	1.3–5.0	4–6

**Table 4:** Flexural test results of sandwich composites.

Sandwich Structures	Load (N)	Flexural Strength (MPa)	Flexural Modulus (GPa)	Load Standard Deviation
**GF/PVC/Elium**	1308	243.75	0.26	18.8
**GF/PVC/Epoxy**	1156	206.25	0.215	12.6
**GF/PET/Elium**	868	165	0.18	16.3
**GF/PET/Epoxy**	845	159.4	0.16	23.03

**Table 5:** Core shear test results of sandwich composites.

Sandwich Structure	Core Shear Ultimate Strength (MPa)	Facing Stress (MPa)
GF/PVC/Elium	0.6	14.8
GF/PVC/Epoxy	0.52	13.12
GF/PET/Elium	0.40	9.8
GF/PET/Epoxy	0.38	9.6

**Table 6:** The experimental and numerical results of GFRP sandwich composites’ peak load (N) under flexural loading.

Sandwich Composite Type	Peak Load (N)		
	EXP.	NUM.	Error
GV/PVC/Elium	1307.9	1378.7	5.4%
GF/PVC/Epoxy	1055.9	1034.5	2%
GF/PET/Elium	867.9	860.5	0.85%
GF/PET/Epoxy	845.2	830.7	1.7%

## Data Availability

Data are contained within the article.
